# Risk Stratification of Long-Term Mortality in Infants with Congenital Diaphragmatic Hernia Using the National Health Insurance Service (NHIS) Data

**DOI:** 10.3390/children13010108

**Published:** 2026-01-12

**Authors:** Hye Ji Han, Min Ji Suh, In Young Choi, Ji Soo Park, Hwan Soo Kim, Hyeon-Jong Yang, Dong In Suh, Eun Lee, Kyung Hoon Kim

**Affiliations:** 1 Department of Pediatrics, Seoul National University Bundang Hospital, Seongnam 13620, Republic of Korea; haeji1993@gmail.com (H.J.H.); gyen97@gmail.com (I.Y.C.); 2 Department of Pediatrics, Seoul National University College of Medicine, Seoul 03080, Republic of Korea; 5d524@snuh.org (M.J.S.); lovinu7@snu.ac.kr (J.S.P.); dongins0@snu.ac.kr (D.I.S.); 3 Department of Pediatrics, College of Medicine, The Catholic University of Korea, Seoul 06591, Republic of Korea; pedhskim@catholic.ac.kr; 4 Department of Pediatrics, Pediatric Allergy and Respiratory Center, Soonchunhyang University College of Medicine, Seoul 04401, Republic of Korea; pedyang@schmc.ac.kr; 5 Department of Pediatrics, Chonnam National University Hospital, Chonnam National University Medical School, Gwangju 61469, Republic of Korea

**Keywords:** congenital diaphragmatic hernia, mortality, survival, population-level risk stratification

## Abstract

**Background:** Congenital diaphragmatic hernia (CDH) is a rare but serious congenital anomaly linked to high mortality rates and significant long-term morbidity. Although numerous prognostic factors for short-term outcomes have been identified through hospital-based studies, data on long-term mortality at the population level are limited. Specifically, nationwide assessments of long-term outcomes for infants with CDH are scarce. This study aimed to estimate the national 5-year all-cause mortality for CDH and to create a population-level risk stratification nomogram utilizing nationwide health insurance claims data. **Methods:** We conducted a retrospective cohort study of infants with CDH using nationwide insurance claims data from 2002 to 2016, allowing for complete 5-year follow-up. We analyzed population-level demographic and clinical proxy variables with Cox proportional hazards models and developed a nomogram for long-term mortality risk stratification. **Results:** Factors such as rural residence, middle-to-high SES, respiratory distress in newborns, and CHD were associated with increased 5-year mortality in infants with CDH. The claims-based nomogram, which incorporated sociodemographic and comorbidity variables, demonstrated moderate discriminatory power (AUC 0.76; C-index 0.78) for population-level risk stratification. **Conclusions:** This nationwide claims-based cohort study provides population-level estimates of 5-year mortality associated with CDH and introduces a nomogram that offers moderate discriminatory ability for long-term risk stratification.

## 1. Background

Congenital diaphragmatic hernia (CDH) is a rare condition characterized by a defect in the diaphragm, allowing abdominal organs to protrude into the thoracic cavity and disrupt normal lung development. The incidence of CDH ranges from approximately 0.8 to 5.7 cases per 10,000 live births, varying across different populations [1,2,3]. In South Korea, previous domestic studies have reported an incidence of CDH of approximately 4 per 10,000 live births (approximately 1 in 2500 live births), which is comparable to estimates reported in other countries [4]. While the exact cause of CDH remains uncertain, it is recognized as multifactorial. Preliminary studies suggest that several maternal factors—such as advanced maternal age, pregestational diabetes, pregestational hypertension, Caucasian ethnicity, tobacco use, and alcohol consumption—may increase the risk of developing CDH [1,5] CDH is associated with significant morbidity and mortality rates, ranging from 20% to 50%, depending on the institution [1,2,3,6]. Recent studies indicate a decline in mortality rates among patients with CDH over time, although these rates remain high [1,6,7,8]. A hospital-based retrospective cohort study identified respiratory insufficiency and concurrent pulmonary hypertension as the leading causes of death in CDH patients, with most fatalities occurring before the age of 17. Factors such as prematurity, low Apgar scores, and a high oxygenation index have been linked to poor short-term outcomes [2,9,10,11]. Notably, the highest mortality rates were observed in newborns aged 2 to 6 days, with rates decreasing as age increases. However, less is known about mortality following intensive care and surgical repair, which may contribute to overall mortality rates [6]. Comorbidities such as cardiac, gastrointestinal, and genitourinary malformations, chromosomal abnormalities, and nutritional deficiencies have been linked to CDH [2,3,12,13,14]. To enhance survival rates for CDH patients, it is essential to gather population-based evidence from large-scale cohorts that can illuminate long-term mortality patterns beyond the neonatal period. Unfortunately, comprehensive nationwide data on long-term outcomes for CDH are still scarce. Consequently, this study aimed to evaluate long-term mortality among children with CDH and to develop a population-level risk stratification nomogram using nationwide health insurance claims data.

## 2. Methods

### 2.1. Study Design and Participants

We conducted a nationwide retrospective cohort study utilizing national health insurance claims data from the Korean National Health Insurance Service (NHIS), which is a mandatory healthcare system that covers approximately 99.4% of South Korea’s 51 million residents. The NHIS database includes detailed patient-level information on demographics, healthcare utilization, prescribed medications, and International Classification of Diseases (ICD) diagnostic codes.

Due to the timing of insurance registration for neonates, which occurs after official birth registration, and the fact that critically ill neonates may be registered only after initial stabilization, data during the earliest neonatal intensive care period are not fully captured in this database. Our study population comprised infants diagnosed with congenital diaphragmatic hernia (CDH) between January 2002 and December 2016, who were followed for up to five years post-diagnosis.

We extracted data on sex, age at diagnosis, socioeconomic status, date of birth, region of residence at birth, comorbidities, and all-cause mortality from administrative records. CDH-related comorbidities were identified using predefined ICD codes based on prior literature. The selection process for the study population, from the customized NHIS cohort to the final analytic sample, is illustrated in Supplementary Figure S1.

### 2.2. Definitions

Congenital diaphragmatic hernia (CDH) was identified using the International Classification of Diseases, 10th Revision (ICD-10) code Q79.0. Prematurity was defined as birth before 37 completed weeks of gestation.

Socioeconomic status (SES) information was sourced from the NHIS database and categorized into 20 levels based on health insurance contribution amounts, with 5% intervals. These levels were then grouped into quartiles: SES 1 (grades 1–5, lowest), SES 2 (grades 6–10, low-to-middle), SES 3 (grades 11–15, middle-to-high), and SES 4 (grades 16–20, highest).

CDH-associated comorbidities were identified using administrative diagnostic codes and included intrauterine growth restriction, respiratory distress of the newborn, secondary pulmonary hypertension, bronchopulmonary dysplasia, congenital respiratory disease, congenital heart disease, neuromuscular disease, and chromosomal abnormalities. Detailed definitions and corresponding ICD codes can be found in Supplementary Table S1.

### 2.3. Statistical Analysis

The primary outcome of the study was all-cause mortality within five years following the diagnosis of CDH. To evaluate the associations between demographic and clinical proxy variables and five-year mortality, we conducted a multivariable logistic regression analysis. A backward elimination approach was utilized for variable selection, resulting in the inclusion of the following variables in the final multivariable model: sex, region of residence, socioeconomic status, prematurity, intrauterine growth restriction, respiratory distress in newborns, bronchopulmonary dysplasia, congenital heart disease, and congenital respiratory disease.

For population-level risk stratification, the dataset was randomly divided into development and internal validation subsets in a 7:3 ratio. To address class imbalance, we applied the synthetic minority over-sampling technique. A nomogram was created to visualize the relative contributions of each variable to mortality risk. Model discrimination was assessed using the area under the receiver operating characteristic curve (AUC) and the concordance index (C-index).

For the longitudinal assessment of mortality, both unadjusted and adjusted hazard ratios were estimated using Cox proportional hazards regression. Kaplan–Meier survival curves were generated based on the presence of major comorbidities. Statistical significance was set at a two-sided *p*-value of less than 0.05, with 95% confidence intervals. All analyses were conducted using SAS Enterprise Guide 7.1^®^ and R version 4.3.2.

## 3. Results

### 3.1. Baseline Characteristics

During the study period from 2002 to 2016, a total of 251,073 individuals aged <18 years were included in the constructed NHIS cohort, among whom 1661 patients diagnosed with CDH were identified for analysis (Supplementary Figure S1). Baseline characteristics are summarized in Table 1. The overall 5-year all-cause mortality rate was 13.0% (n = 216). Male patients comprised 59.3% of the cohort (n = 985), with 118 deaths (12.0%) occurring within 5 years. Prematurity (defined as less than 37 weeks of gestation) was noted in 26 patients (1.6%). Although CDH was more prevalent among urban residents (48.7%, n = 808), the 5-year mortality rate was higher in rural areas compared to cities (17.8% vs. 11.5%). Among patients classified in the lowest socioeconomic status (SES) group (n = 1154, 69.5%), 100 patients (8.7%) died during follow-up. Congenital heart disease (CHD) was the most common comorbidity, present in 38 patients (2.3%).

### 3.2. Primary Analysis of 5-Year Mortality Risk Stratification

Multivariable logistic regression was conducted to evaluate factors associated with 5-year mortality in patients with congenital diaphragmatic hernia (CDH) (Table 2). Living in rural areas and having a middle-to-high socioeconomic status (SES) were significantly linked to higher mortality when compared to urban residence (adjusted OR, 4.68; 95% CI, 3.55–6.21) and the lowest SES group (adjusted OR, 2.04; 95% CI, 1.43–2.93), respectively. Additionally, respiratory distress in newborns (adjusted OR, 1.16; 95% CI, 1.02–1.32) and congenital heart disease (adjusted OR, 1.11; 95% CI, 1.01–1.23) were associated with increased mortality.

**Figure 1:**
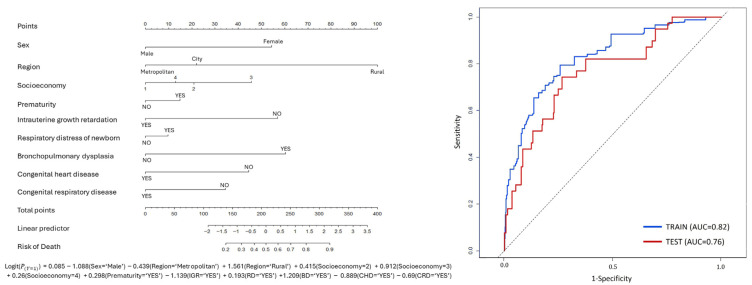
Nomogram for population-level risk stratification of 5-year mortality in patients with congenital diaphragmatic hernia and its validation using a receiver operating characteristic (ROC) curve.

A nomogram was created based on a multivariable model to summarize the associations between sociodemographic and clinical factors and 5-year mortality in patients with congenital diaphragmatic hernia (CDH) (Figure 1). In the nomogram, each variable is assigned a score on the ‘Points’ axis based on its relative contribution to the model. The total score, obtained by summing the individual scores, indicates the estimated probability of death. The ‘Linear Predictor’ represents the combined effect of the regression coefficients for the variables included in the model. Key factors related to CDH, such as prematurity, respiratory distress in newborns, bronchopulmonary dysplasia, congenital heart disease, congenital respiratory disease, intrauterine growth restriction, region of residence at birth, and socioeconomic status, were incorporated into the nomogram. This model allows for the estimation of relative mortality risk across different population subgroups. Internal validation of the model showed moderate discriminatory performance, with a concordance index (C-index) of 0.78 and an area under the receiver operating characteristic curve (AUC) of 0.76.

### 3.3. Survival Analysis According to Comorbidities

Multivariable Cox proportional hazards regression analysis did not reveal statistically significant associations between most prespecified comorbidities and 5-year all-cause mortality in patients with CDH (Table 3). Although secondary pulmonary hypertension was associated with a higher hazard of mortality (HR, 4.33; 95% CI, 1.38–13.54), this association did not achieve statistical significance after multivariable adjustment.

**Figure 2:**
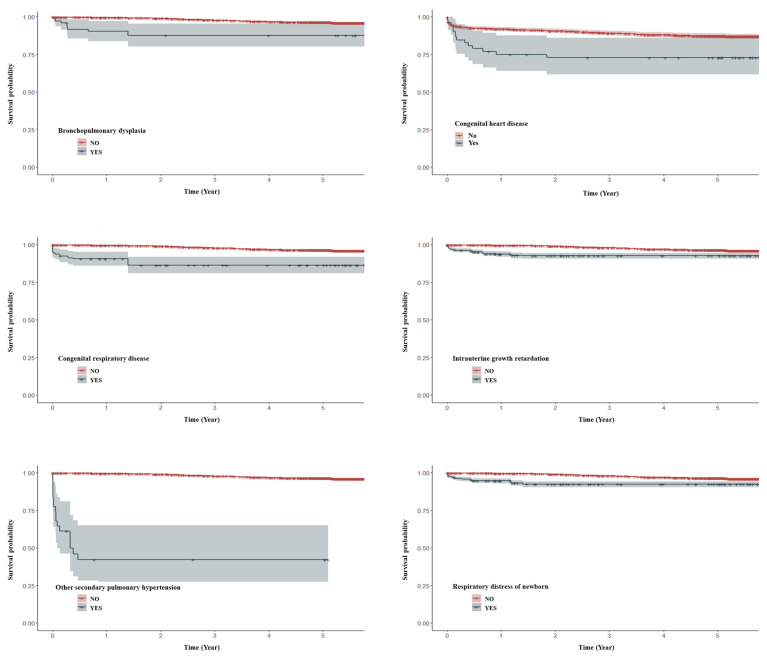
Kaplan–Meier survival curves for 5-year survival after congenital diaphragmatic hernia diagnosis, stratified by the presence of CDH-related comorbidities.

Kaplan–Meier survival analysis was conducted to compare 5-year survival rates following a diagnosis of congenital diaphragmatic hernia between patients with and without CDH-related clinical conditions, such as bronchopulmonary dysplasia, congenital heart disease, congenital respiratory disease, intrauterine growth restriction, respiratory distress of the newborn, and other secondary pulmonary hypertension. Among these groups, patients with other secondary pulmonary hypertension showed consistently lower survival probabilities over the 5-year follow-up period compared to those without this condition (Figure 2).

## 4. Discussion

In this nationwide, population-based cohort study utilizing national health insurance claims data, we estimated the 5-year all-cause mortality rate for children with congenital diaphragmatic hernia (CDH) in Korea to be 13.0%. Factors associated with higher long-term mortality included being born in rural areas, having a middle-to-high socioeconomic status (SES), and the presence of certain comorbidities, such as respiratory distress of the newborn (RD) and congenital heart disease (CHD). Additionally, we developed a claims-based nomogram to aid in population-level risk stratification of 5-year mortality. Rather than functioning as a bedside clinical prediction tool, this model offers an epidemiological overview of long-term mortality patterns and sociodemographic disparities among children with CDH at the national level.

CDH is a severe congenital condition linked to significant morbidity and mortality, though survival rates have improved with advancements in perinatal and neonatal care. Previous hospital-based studies mainly concentrated on early mortality and short-term outcomes, identifying key prognostic factors such as respiratory insufficiency, pulmonary hypertension, prematurity, and low Apgar scores. In contrast, long-term mortality after stabilization and discharge from neonatal intensive care units has been less thoroughly researched. As the current cohort predominantly includes survivors beyond the immediate perinatal period, the observed 5-year mortality rate is lower than that reported in hospital-based cohorts, yet it remains clinically significant since early in-hospital deaths were not included.

A notable finding from our population-level analysis is the substantial disparity in mortality based on region of residence and socioeconomic status. The nearly five-fold increase in mortality risk for infants born in rural areas (Adjusted OR 4.68) underscores a crucial gap in specialized long-term follow-up care. Unlike hospital-based studies that prioritize immediate surgical success, our results highlight the urgent need to establish a ‘nationwide care network’ to ensure that survivors in underserved regions have equal access to pediatric subspecialty care and rehabilitation.

Furthermore, the independent association of respiratory distress and congenital heart disease with 5-year mortality indicates that these comorbidities create a lasting physiological burden. Even after successful diaphragmatic repair, these underlying conditions can heighten the risk of late-onset complications, requiring extended and careful clinical monitoring.

In this study, CHD was the most commonly observed comorbidity among children with CDH, followed by intrauterine growth restriction, RD, congenital respiratory diseases, secondary pulmonary hypertension, bronchopulmonary dysplasia, and chromosomal abnormalities. This pattern aligns with previous reports that identified cardiac anomalies, pulmonary hypertension, and chromosomal abnormalities as frequent coexisting conditions in CDH. Although the overall prevalence of comorbidities was higher in children who died compared to survivors, most were not independently linked to long-term mortality after multivariable adjustment. This suggests that the impact of certain comorbidities may be more significant during the early postnatal period, whereas their effects on long-term outcomes after discharge might diminish.

In the time-to-event analysis, secondary pulmonary hypertension showed a numerically higher hazard of mortality, though this association lacked statistical significance after adjustment. Caution is advised in interpreting this result, as early in-hospital deaths and detailed physiological data were not accessible in the claims database. Previous single-center studies have identified pulmonary hypertension as a strong predictor of early mortality in CDH, emphasizing the importance of disease severity during the neonatal period. The current findings suggest that, among survivors who have stabilized, the long-term effects of individual comorbidities may differ from their short-term prognostic significance.

Several clinical prediction models for CDH have been developed using hospital-based cohorts, incorporating variables such as birth weight, Apgar scores, pulmonary hypertension, liver herniation, and major cardiac or chromosomal anomalies. These models have shown high discriminative ability for short-term or in-hospital mortality. However, they rely on detailed clinical and physiological data that are often unavailable in administrative datasets and frequently do not consider sociodemographic factors or long-term outcomes post-discharge. In contrast, the present study emphasizes population-level risk stratification using nationwide longitudinal data, which complements existing hospital-based research by offering insights into long-term mortality patterns in a real-world context.

Notably, disparities in mortality were observed based on region of residence at birth and SES. Sociodemographic variables significantly contributed to the overall risk stratification in the claims-based nomogram, at times even more so than clinical proxy variables. These results align with previous reports indicating that survival in CDH may be affected by broader social and healthcare system factors, including access to specialized care, regional healthcare infrastructure, and socioeconomic resources. While the underlying mechanisms were not directly examined in this study, the observed associations highlight the potential significance of sociodemographic context in long-term outcomes.

Several limitations of this study should be acknowledged. First, the reliance on insurance claims data restricted access to detailed clinical information, such as Apgar scores, anatomic subtypes of CDH, physiological severity measures, and specific perinatal management strategies. In particular, variables like surgical details and extracorporeal membrane oxygenation (ECMO) were only available for a limited number of cases, making reliable incorporation into the analysis challenging. Second, since early in-hospital deaths were not recorded in the claims database, the study population predominantly consists of children who survived the immediate neonatal period.

As a result, the estimated 5-year mortality reflects long-term outcomes for survivors after discharge, rather than overall mortality from birth. Additionally, cause-specific mortality could not be accurately determined from the available data, so all-cause mortality was used as the primary outcome measure. Oversampling techniques were employed during the development of the nomogram, which may have affected the relative impact of individual predictors. Furthermore, temporal changes in perinatal and neonatal care practices over the extended study period were not explicitly modeled, potentially contributing to variations in outcomes.

Despite these limitations, this study capitalizes on the strengths of a nationwide cohort with nearly complete population coverage and long-term follow-up. By providing population-level estimates of 5-year mortality and highlighting sociodemographic disparities, this claims-based analysis complements hospital-based clinical studies and may inform future research and policy discussions on long-term outcomes and health equity in children with CDH.

## 5. Conclusions

This nationwide claims-based cohort study offers population-level estimates of 5-year all-cause mortality for children with congenital diaphragmatic hernia. By integrating sociodemographic and administrative clinical proxy variables, we created a nomogram for risk stratification of long-term mortality at the population level. While the study is limited by the lack of detailed clinical information, it provides valuable epidemiologic insights into long-term mortality trends and sociodemographic disparities in CDH across the nation.

## Figures and Tables

**Table 1:** Clinical characteristics of the patients.

Characteristics	Survival	Death	Total
	(n = 1445)	(n = 216)	(n = 1661)
Sex–male, n (%)	867 (62.06)	118 (55.66)	985 (59.3)
Prematurity—<37 weeks, n (%)	1 (0.07)	25 (11.57)	26 (1.57)
Region of residence at birth, n (%)			
City	715 (49.48)	93 (43.06)	808 (48.65)
Metropolitan	328 (22.70)	36 (16.67)	364 (21.91)
Rural	402 (27.82)	87 (40.28)	489 (29.44)
Socioeconomic status, n (%)			
lowest	1054 (72.94)	100 (46.30)	1154 (69.48)
2nd quartile	170 (11.76)	41 (18.98)	211 (12.7)
3rd quartile	170 (11.76)	60 (27.78)	230 (13.85)
highest	51 (3.53)	15 (6.94)	66 (3.97)
Comorbidities—yes, n (%)			
Respiratory distress of newborn	1 (0.07)	24 (11.11)	25 (1.51)
Intrauterine growth restriction	1 (0.07)	29 (13.43)	30 (1.81)
Bronchopulmonary dysplasia	0 (0)	5 (2.31)	5 (0.30)
Congenital heart disease	3 (0.21)	35 (16.20)	38 (2.29)
Congenital respiratory disease	0 (0)	14 (6.48)	14 (0.84)
Neuromuscular disease	0 (0)	0 (0)	0 (0)
Chromosomal abnormalities	0 (0)	5 (2.31)	5 (0.3)
Secondary pulmonary hypertension	0 (0)	11 (5.09)	11 (0.66)

Percentages in the Survival and Death columns are calculated based on the column totals. (n = 1445 and n = 216, respectively). Percentages in the Total column are calculated based on the overall study population (n = 1661). The overall 5-year all-cause mortality rate in the study cohort was 13.0% (216/1661).

**Table 2:** Unadjusted and adjusted odds ratios for mortality within 5 years.

Variables	Unadjusted Odds Ratio	Adjusted Odds Ratio
	(95% CI)	(95% CI)
Sex–male	0.97 (0.94–1.00)	0.98 (0.94–1.01)
Prematurity—<37 weeks	1.08 (0.96–1.21)	1.05 (0.81–1.37)
Regions of residence at birth—rural	5.40 (4.14–7.07) ^†^	4.68 (3.55–6.21) ^†^
Socioeconomic status—3rd quartile	1.86 (1.35–2.56) ^†^	2.04 (1.43–2.93) ^†^
Respiratory distress of newborn	1.01 (0.94–1.10)	1.16 (1.02–1.32) ^†^
Intrauterine growth restriction	0.97 (0.90–1.05)	0.95 (0.75–1.20)
Bronchopulmonary dysplasia	1.13 (0.89–1.43)	1.03 (0.80–1.34)
Congenital heart disease	1.03 (0.96–1.10)	1.11 (1.01–1.23) ^†^
Congenital respiratory disease	0.98 (0.86–1.10)	0.96 (0.83–1.12)
Neuromuscular disease	0.88 (0.71–1.08)	0.86 (0.69–1.06)
Chromosomal abnormalities	1.07 (0.87–1.32)	1.12 (0.85–1.48)
Secondary pulmonary hypertension	0.56 (0.44–0.71) ^†^	1.18 (0.93–1.50)

Multivariate analysis by logistic regression was performed on the dichotomous outcome represented as mortality within 5 years. The odds ratios for the unadjusted analysis were calculated, and then adjusted for sex, prematurity, region of residence at birth, socioeconomic status, and other comorbidities. Statistical significance was indicated by crosses (†), and a 95% confidence interval was used for the analysis. CI refers to confidence interval.

**Table 3:** Unadjusted and adjusted hazard ratios for mortality within 5 years.

Variables	Unadjusted Hazard Ratio	Adjusted Hazard Ratio
	(95% CI)	(95% CI)
Respiratory distress of newborn	1.42 (0.94–2.44)	1.87 (0.78–4.46)
Intrauterine growth restriction	0.95 (0.47–1.92)	0.48 (0.18–1.32)
Bronchopulmonary dysplasia	2.10 (0.52–8.46)	2.16 (0.46–10.07)
Congenital heart disease	1.42 (0.83–2.44)	1.28 (0.65–2.50)
Congenital respiratory disease	0.89 (0.28–2.78)	0.72 (0.22–2.38)
Secondary pulmonary hypertension	4.33 (1.38–13.54) ^†^	3.33 (0.98–11.28)

Multivariate survival analysis was performed using Cox proportional hazard regression. The unadjusted hazard ratios with confidence intervals were calculated and adjusted for sex, prematurity, region of residence at birth, socioeconomic status, and other comorbidities. Statistical significance was indicated with crosses (†), and a 95% confidence interval was chosen for analysis. CI refers to confidence interval.

## Data Availability

The datasets analyzed in this study are available from the corresponding author upon reasonable request.

## Supplementary Materials

The following supporting information can be downloaded at: https://www.mdpi.com/article/10.3390/children13010108/s1, Table S1: The International Classification of Diseases codes for comorbidities in patients with congenital diaphragmatic hernia. The comorbidities are listed with the number of patients affected and the International Classification of Diseases codes: ICD-10, International Classification of Diseases 10th Revision; Figure S1: Flow diagram illustrating the identification of patients with congenital diaphragmatic hernia within the National Health Insurance Service (NHIS) cohort.

## Abbreviations

CDH	Congenital diaphragmatic hernia
NHIS	National Health Insurance Service
ICD	International Classification of Diseases
SES	socioeconomic status
RD	respiratory distress of newborn
CHD	congenital heart disease
AUC	area under the receiver operating curve
C-index	concordance index
HR	hazard ratio
CI	confidence interval
OR	odds ratio
