# Supplementary material for: Risk Stratification of Long-Term Mortality in Infants with Congenital Diaphragmatic Hernia Using the National Health Insurance Service (NHIS) Data
Source: Children (Basel). 2026 Jan 12;13(1):108. doi: 10.3390/children13010108 (PMC12839643; doi:10.3390/children13010108)
Supplement: Supplementary file 1 [file children-13-00108-s001.zip › children-4052212-supplementary/sup_rev/Supplementary table S1_fin.docx]

**Supplementary table S1. The International Classification of Diseases codes of comorbidities with congenital diaphragmatic hernia.**

| **Categories** | **ICD-10**  **Code** | **No.** **of**  **patients** |
| --- | --- | --- |
| **Secondary** **pulmonary** **hypertension** |  |  |
| Other secondary pulmonary hypertension | I27.2 | 11 |
| **Respiratory** **distress** **of** **newborn** |  |  |
| Respiratory distress syndrome of newborn | P22.0 | 21 |
| Transient tachypnoea of newborn | P22.1 | 1 |
| Other respiratory distress of newborn | P22.8 | 1 |
| Respiratory distress of newborn, unspecified | P22.9 | 3 |
| **Intrauterine** **growth** **retardation** |  |  |
| Small for gestational age | P05.1 | 1 |
| **Bronchopulmonary** **displasia** |  |  |
| Bronchopulmonary dysplasia originating in the perinatal period | P27.1 | 5 |
| **Congenital** **heart** **disease** |  |  |
| **Congenital** **malformations** **of** **cardiac** **chambers** **and** **connections** | **Q20** |  |
| Double outlet right ventricle | Q20.1 | 1 |
| Double inlet ventricle | Q20.4 | 2 |
| Isomerism of atrial appendages | Q20.6 | 1 |
| **Congenital** **malformations** **of** **cardiac** **septa** | **Q21** |  |
| Ventricular septal defect | Q21.0 | 7 |
| Perimembranous Ventricular septal defect | Q21.01 | 2 |
| Other ventricular septal defect | Q21.08 | 3 |
| Ventricular septal defect, unspecified | Q21.09 | 2 |
| Atrial septal defect | Q21.1 | 12 |
| Patent or persistent foramen ovale | Q21.10 | 3 |
| Other atrial septal defect | Q21.18 | 4 |
| Atrial septal defect, unspecified | Q21.19 | 2 |
| Atrioventricular septal defect | Q21.2 | 2 |
| Tetralogy of Fallot | Q21.3 | 2 |
| **Congenital** **malformations** **of** **aortic** **and** **mitral** **valves** | **Q23** |  |
| Congenital mitral insufficiency | Q23.3 | 1 |
| Hypoplastic left heart syndrome | Q23.4 | 2 |
| **Other** **congenital** **malformations** **of** **heart** | **Q24** |  |
| Dextrocardia | Q24.0 | 1 |
| Other specified congenital malformations of heart | Q24.8 | 2 |
| Congenital malformation of heart, unspecified | Q24.9 | 2 |
| **Congenital** **malformations** **of** **great** **arteries** | **Q25** |  |
| Patent ductus arteriosus | Q25.0 | 15 |
| Coarctation of aorta | Q25.1 | 2 |
| Other congenital malformations of aorta | Q25.4 | 1 |
| **Congenital** **malformations** **of** **great** **veins** | **Q26** |  |
| Total anomalous pulmonary venous connection | Q26.2 | 1 |
| **Other** **congenital** **malformations** **of** **peripheral** **vascular** **system** | **Q27** |  |
| Congenital absence and hypoplasia of umbilical artery | Q27.0 | 1 |
| **Congenital** **respiratory** **disease** |  |  |
| **Congenital** **malformations** **of** **lung** | **Q33** |  |

The comorbidities associated with congenital diaphragmatic hernia were listed with the International Classification of Diseases codes.
