# Supplementary material for: Risk Stratification of Long-Term Mortality in Infants with Congenital Diaphragmatic Hernia Using the National Health Insurance Service (NHIS) Data
Source: Children (Basel). 2026 Jan 12;13(1):108. doi: 10.3390/children13010108 (PMC12839643; doi:10.3390/children13010108)
Supplement: Supplementary file 1 [file children-13-00108-s001.zip › children-4052212-supplementary/sup_rev/Supplementary table S1_fin.pdf]

Supplementary table S1. The International Classification of Diseases codes of comorbidities with congenital diaphragmatic hernia.

Categories	ICD-10 Code	No. of patients
**Secondary pulmonary hypertension**		
Other secondary pulmonary hypertension	I27.2	11
**Respiratory distress of newborn**		
Respiratory distress syndrome of newborn	P22.0	21
Transient tachypnoea of newborn	P22.1	1
Other respiratory distress of newborn	P22.8	1
Respiratory distress of newborn, unspecified	P22.9	3
**Intrauterine growth retardation**		
Small for gestational age	P05.1	1
**Bronchopulmonary displasia**		
Bronchopulmonary dysplasia originating in the perinatal period	P27.1	5
**Congenital heart disease**		
**Congenital malformations of cardiac chambers and connections**	**Q20**	
Double outlet right ventricle	Q20.1	1
Double inlet ventricle	Q20.4	2
Isomerism of atrial appendages	Q20.6	1
**Congenital malformations of cardiac septa**	**Q21**	
Ventricular septal defect	Q21.0	7
Perimembranous Ventricular septal defect	Q21.01	2
Other ventricular septal defect	Q21.08	3
Ventricular septal defect, unspecified	Q21.09	2
Atrial septal defect	Q21.1	12
Patent or persistent foramen ovale	Q21.10	3
Other atrial septal defect	Q21.18	4
Atrial septal defect, unspecified	Q21.19	2
Atrioventricular septal defect	Q21.2	2
Tetralogy of Fallot	Q21.3	2
**Congenital malformations of aortic and mitral valves**	**Q23**	
Congenital mitral insufficiency	Q23.3	1
Hypoplastic left heart syndrome	Q23.4	2
**Other congenital malformations of heart**	**Q24**	
Dextrocardia	Q24.0	1
Other specified congenital malformations of heart	Q24.8	2
Congenital malformation of heart, unspecified	Q24.9	2
**Congenital malformations of great arteries**	**Q25**	
Patent ductus arteriosus	Q25.0	15
Coarctation of aorta	Q25.1	2
Other congenital malformations of aorta	Q25.4	1
**Congenital malformations of great veins**	**Q26**	
Total anomalous pulmonary venous connection	Q26.2	1

**Other congenital malformations of peripheral vascular system**	**Q27**	
Congenital absence and hypoplasia of umbilical artery	Q27.0	1
**Congenital respiratory disease**		
**Congenital malformations of lung**	**Q33**	

The comorbidities associated with congenital diaphragmatic hernia were listed with the International Classification of Diseases codes.
