# Supplementary material for: Risk Stratification of Long-Term Mortality in Infants with Congenital Diaphragmatic Hernia Using the National Health Insurance Service (NHIS) Data
Source: Children (Basel). 2026 Jan 12;13(1):108. doi: 10.3390/children13010108 (PMC12839643; doi:10.3390/children13010108)
Supplement: Supplementary file 1 [file children-13-00108-s001.zip › children-4052212-supplementary/sup_rev/title page_final.docx]

**Title**: Risk stratification of long-term mortality in infants with congenital diaphragmatic hernia using the national health insurance service (NHIS) Data

**Authors**: Hye Ji Han^1*^ Min Ji Suh^2*^, In Young Choi^1^, Ji Soo Park^2^, Hwan Soo Kim^3^, Hyeon-Jong Yang^4^, Dong In Suh^2^, Eun Lee^5‡^, Kyung Hoon Kim^1,2‡^

**Affiliations**:

^1^Department of Pediatrics, Seoul National University Bundang Hospital, Seongnam, Korea

^2^Department of Pediatrics, Seoul National University College of Medicine, Seoul, Korea

^3^Department of Pediatrics, College of Medicine, The Catholic University of Korea, Seoul, Korea

^4^Department of Pediatrics, Pediatric Allergy and Respiratory Center, Soonchunhyang University School of Medicine, Korea

^5^Department of Pediatrics, Chonnam National University Hospital, Chonnam National University Medical School, Gwangju, Korea

* these authors contributed equally to this work

‡ these authors contributed equally to this work.

**Address for Correspondence**:

Kyunghoon Kim, MD, PhD

Department of Pediatrics, Seoul National University College of Medicine,

Seoul National University Bundang Hospital, Seongnam, Republic of Korea, 13620

E-mail: [journey237@snu.ac.kr](mailto:journey237@snu.ac.kr)

Eun Lee, MD, PhD

Department of Pediatrics, Chonnam National University Medical School,

Chonnam National University Hospital, Gwangju, Republic of Korea, 61469

E-mail: unelee@daum.net
