# Supplementary figures and images for: Risk Stratification of Long-Term Mortality in Infants with Congenital Diaphragmatic Hernia Using the National Health Insurance Service (NHIS) Data
Source: Children (Basel). 2026 Jan 12;13(1):108. doi: 10.3390/children13010108 (PMC12839643; doi:10.3390/children13010108)

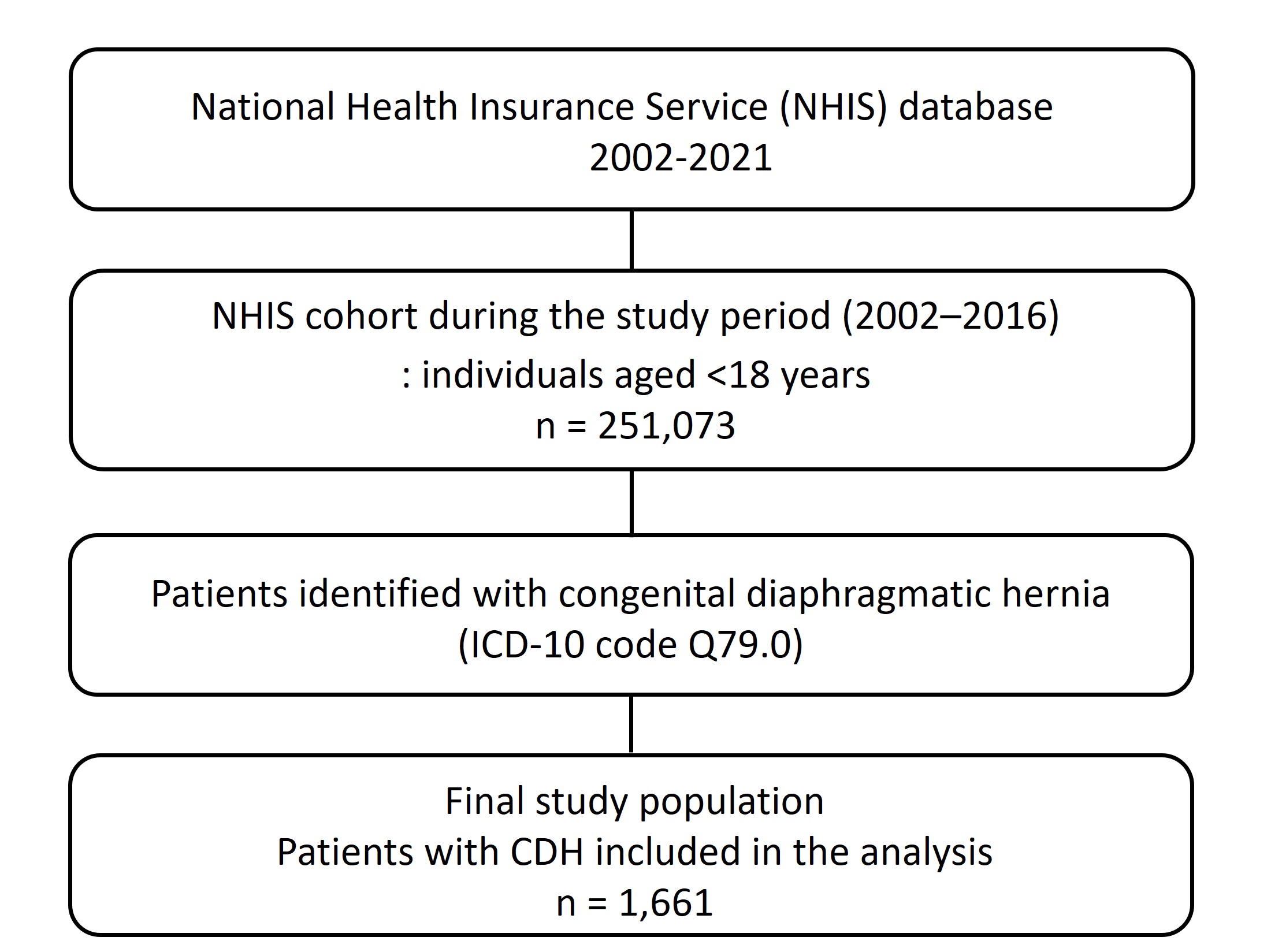

Supplement: Supplementary file 1 [file children-13-00108-s001.zip › children-4052212-supplementary/supplementary_figure1_fin.jpg]
